# Overexpression of synuclein-γ predicts lack of benefit from radiotherapy for breast cancer patients

**DOI:** 10.1186/s12885-016-2750-y

**Published:** 2016-09-05

**Authors:** Li Min, Cheng Zhang, Ruolan Ma, Xiaofan Li, Hua Yuan, Yihao Li, Ruxuan Chen, Caiyun Liu, Jianping Guo, Like Qu, Chengchao Shou

**Affiliations:** 1 Department of Biochemistry and Molecular Biology, Key Laboratory of Carcinogenesis and Translational Research (Ministry of Education), Peking University Cancer Hospital & Institute, Beijing, 100142 China; 2 Department of Biostatistics and Computational Biology, Dana-Farber Cancer Institute, Harvard School of Public Health, Boston, MA 02115 USA; 3 Department of Thoracic Surgery, Key Laboratory of Carcinogenesis and Translational Research (Ministry of Education), Peking University Cancer Hospital & Institute, Beijing, 100142 China; 4 Department of Radiotherapy, Key Laboratory of Carcinogenesis and Translational Research (Ministry of Education), Peking University Cancer Hospital & Institute, Beijing, 100142 China; 5 Key Laboratory of Carcinogenesis and Translational Research (Ministry of Education), Breast Center, Peking University Cancer Hospital & Institute, Beijing, 100142 China; 6 Department of Biostatistics, UCLA School of Public Health, Los Angeles, CA 90024 USA; 7 Peking Union Medical College, Chinese Academy of Medical Sciences, Beijing, 100730 China

**Keywords:** Synuclein-γ, Radiotherapy, Prognosis, Breast cancer

## Abstract

**Background:**

Although radiotherapy following mastectomy was demonstrated to reduce the recurring risk and improve the prognosis of patients with breast cancer, it is also notorious for comprehensive side effects, hence only a selected group of patients can benefit. Therefore, the screening of molecular markers capable of predicting the efficacy of radiotherapy is essential.

**Methods:**

We have established a cohort of 454 breast cancer cases and selected 238 patients with indications for postoperative radiotherapy. Synuclein-γ (SNCG) protein levels were assessed by immunohistochemistry, and SNCG status was retrospectively correlated with clinical features and survival in patients treated or not treated with radiotherapy. Gene Set Enrichment Analysis (GSEA) and survival analysis for online datasets were also performed for further validation.

**Results:**

Among patients that received radiotherapy (82/238), those demonstrating positive SNCG expression had a 55.0 month shorter median overall survival (OS) in comparison to those demonstrating negative SNCG expression (78.4 vs. 133.4 months, log rank *χ*^*2*^ = 16.13; *p* < 0.001). Among the patients that received no radiotherapy (156/238), SNCG status was not correlated with OS (log rank *χ*^*2*^ = 2.40; *p* = 0.121). A COX proportional hazard analysis confirmed SNCG as an independent predictor of OS, only for patients who have received radiotherapy. Similar results were also obtained for distant metastasis-free survival (DMFS). A GSEA analysis indicated that SNCG was strongly associated with genes related to a radiation stress response. A survival analysis was performed with online databases consisting of breast cancer, lung cancer, and glioblastoma and further confirmed SNCG’s significance in predicting the survival of patients that have received radiotherapy.

**Conclusion:**

A positive SNCG may serve as a potential marker to identify breast cancer patients who are less likely to benefit from radiotherapy and may also be extended to other types of cancer. However, the role of SNCG in radiotherapy response still needs to be further validated in randomized controlled trials prior to being exploited in clinical practice.

**Electronic supplementary material:**

The online version of this article (doi:10.1186/s12885-016-2750-y) contains supplementary material, which is available to authorized users.

## Background

Breast cancer is the most frequently diagnosed cancer among females worldwide [1]. In more developed countries like the U.S., breast cancer death rates have slowly decreased by 1.4 % per year [2, 3]; however, in less developed areas, both of the incidence rate and mortality rate of breast cancer are still raising [1]. In 2013, breast cancer accounted for 25 % of total cancer cases and 15 % of cancer-related deaths worldwide [1]. For decades, surgical removal of the primary tumor has been the major therapeutic option [4, 5], and the addition of adjuvant radiotherapy based on a risk of recurrence and metastasis has been found to significantly improve the overall prognosis. Currently, adjuvant radiotherapy after mastectomy has been widely accepted as the gold standard of care for patients with tumors > 5 cm in size, 4 or more positive lymph nodes, or positive margins [6]. However, radiotherapy is also associated with potential long-term side effects and radiation oncologists have to be highly selective of patients and administer radiation treatments with extreme caution [6, 7]. Despite such precautions, not every patient subjected to radiotherapy can particularly benefit from it. Thus, biomarkers capable of predicting radiotherapeutic efficacy would largely strengthen current clinical options by providing instructions for appropriate risk evaluation and treatment plan selection.

Synuclein-γ (SNCG) was first identified as breast cancer–specific gene 1 (BCSG1), and was isolated from cDNA libraries of breast carcinoma in the 1990s [8, 9]. SNCG is highly expressed in advanced and metastatic breast tumors but not in normal breast epithelium tissues. In breast cancer cells, SNCG protein impairs cell cycle checkpoints [10, 11], confers chemoresistance [12, 13], and enhances metastasis in nude mice [14]. Although the detailed mechanism is not fully understood, SNCG’s role in the oncogenesis-related Akt and mTOR pathways [15] and the neural development-related PPARγ pathway [16] are noteworthy and worth further investigation. The poor overall SNCG-related prognosis in breast cancer has been reported by two independent studies [17, 18]. Moreover, SNCG was overexpressed in other cancerous tissues and this overexpression was a prediction of poor prognosis in several types of cancer [17–22]. Nevertheless, the relationship between SNCG expression and radiotherapeutic efficacy remains to be elucidated.

The aim of this study is to explore the impact of SNCG expression on the prognosis as well as multiple clinical manifestations of breast cancer patients treated with radiotherapy. Surgically resected specimens from breast cancer patients as well as expression profiling datasets from online repositories were simultaneously analyzed. SNCG expression and its relationship with pathological parameters were investigated on both protein and transcript levels, and high SNCG expression were suggested to be an indication of fewer radiotherapeutic benefits. Furthermore, our finding was also validated by analysis performed in two online datasets of different cancer types with radiotherapy information. In conclusion, this study has revealed the prospective value of SNCG expression in predicting whether breast cancer patients could benefit from radiotherapy, and could further potentially be used as a significant parameter for cancer adjuvant treatment.

## Methods

### Patient selection

A cohort of 454 invasive breast cancer patients that received radical or modified radical mastectomy between the years of 1996 and 2002 in the Breast Center at the Peking University Cancer Hospital & Institute. The project was approved and supervised by the research ethics committee of Peking University Cancer Hospital & Institute. Written informed consents were obtained from all participants. Patients with indications for postoperative radiotherapy were recruited: patients with T3/4 tumors (i.e. tumor size > 5 cm in size, or positive margins), patients with four or more positive lymph nodes, T1/2 patients with one to three positive nodes and other risk factors of recurrence (i.e. ≤ 40 years old, hormone receptor-negative, HER2 positive, incomplete lymph node dissection or more than 20 % positive nodes). The presence of ER and PR was evaluated using the charcoal-dextran method. ER and PR values of more than 10 fmol/mg were considered positive. Status of HER2 was assessed by IHC with a rabbit polyclonal antibody (DAKO A0485; 1:250 dilution), and scored by the Diagnostic Pathological Department, Peking University Cancer Hospital. Eight fields were randomly selected in each slide and slides were counted under a Nikon microscope at 200× amplification [17]. Among the 454 breast cancer patient cohort, 238 of the cases with indications for postoperative radiotherapy were selected while only 82 of them had been treated with radiotherapy. No patients involved in this study have received neoadjuvant chemotherapy.

### Radiotherapy treatment

Overall, there were 238 patients with indications for postoperative radiotherapy that were selected. The selected patients were aged from 25 to 81 years (median 52 years). 82 of them were typically treated with standard radiotherapy in 25 fractions (50 Gy at 2 Gy per fraction, 5 fractions per week), and ensured that the radiotherapy dose was actually delivered to the CTV (clinical target volume) with 6 MV photons or electron beam. The remaining 156 patients had not been subjected to radiotherapy.

### Clinical samples handling

Surgically resected tissue specimens were used in this study. Formalin fixed, paraffin-embedded breast cancer tissue specimens from the above 238 patients were obtained from the Breast Center at Peking University Cancer Hospital & Institute. The study was approved and supervised by the Medical Ethics Committee of Peking University Cancer Hospital & Institute and each patient had given formal consent. All specimens were taken before the onset of chemotherapy or hormonal treatment. The total period of follow-up was 60–192 months with a median period of 127 months.

### Immunohistochemical staining

Specimens were cut into 5 μm sections. After baking at 60 °C overnight, sections were dewaxed and rehydrated through xylene and alcohol series. Antigen retrieval was performed via microwave cooking in ethylene diamine tetra acetic acid (pH 8.0, Zymed) for 20 min. Endogenous peroxidase activity was blocked by incubation in 3 % hydrogen peroxide for 10 min at room temperature. Non-specific binding was blocked with 10 % goat serum. Then slides were subjected to overnight incubation at 4 °C with anti-SNCG monoclonal antibody generated in our laboratory [17]. After incubation with a biotin-conjugated secondary anti-mouse antibody for 30 min and 3 washes with phosphate-buffered saline with 0.1 % Tween-20, slides were treated with diaminobenzidine (DAB) working solution at room temperature for 3–10 min, and then washed in distilled water and counterstained with hematoxylin. The negative control was prepared by replacing the SNCG antibody with non-immune IgG in a randomly selected breast cancer tissue slide, and the positive control was prepared with SNCG antibody in a known SNCG positive breast cancer tissue slide which had been proved in a previous study [17].

### IHC grading system

All of the samples were independently inspected under a light microscope (APPLIED IMAGING at 200×) by two experienced pathologists. Both the percentage of positive cells and the intensity of staining in 10 randomly chosen microscopic fields were evaluated. According to our previous publications, the grading system was based on a 4-value classification scale as follows: the area of staining was graded as <10 % (0) or >10 % (1) of all cancer cells stained within the section; intensity of staining was graded as none (0), weak (1), moderate (2) or strong (3). The final grade was obtained by adding area grade and intensity grade together, and final grade ≥ 3 was defined as positive [20, 23].

### Validating analysis

EBI ArrayExpress dataset *E-TABM-158* and NCBI GEO dataset *GSE1456* are two online breast cancer datasets with radiotherapy information [24, 25]. Both of the two datasets with their supplementary clinical information were downloaded and used for validating analysis. A pearson correlation analysis was performed to assess the gene-gene expression correlation. A hierarchical clustering was used to distinguish different subgroups according to expression level of given genes. Gene Set Enrichment Analysis (GSEA) was performed to evaluate correlation between SNCG expression and two radiation stress response gene sets [26, 27]. Lung cancer dataset *CaArray* and glioblastoma dataset *GSE13041* with radiotherapy information were also downloaded and used for validating analysis [28–30].

### Statistical analysis

Since the populations of stage II patients in the radiotherapy subgroup were too small to perform a separate multivariate analysis, we combined the samples in stage II and III to make it sufficient for statistics. All statistical analyses were performed using the R 3.1.2 software (www.r-project.org). Correlations that were made between the SNCG expression and clinicopathologic characteristics were tested by the Pearson *χ*^*2*^ test. The Kaplan-Meier curve was used to evaluate overall survival (OS) and distant metastasis-free survival (DMFS) rates, and differences were tested by log-rank test. The COX proportional hazard model was used for multivariate analysis. Hazard ratios (HR) and 95 % confidence interval (CI) were calculated. All statistical analyses were 2-sided, and a *p* value less than 0.05 were considered statistically significant. For false discovery rate (FDR) analysis, a cutoff of 0.25 was selected according to GSEA’s suggestion [26].

## Results

### Association of SNCG expression and clinicopathologic features

**Fig. 1:**
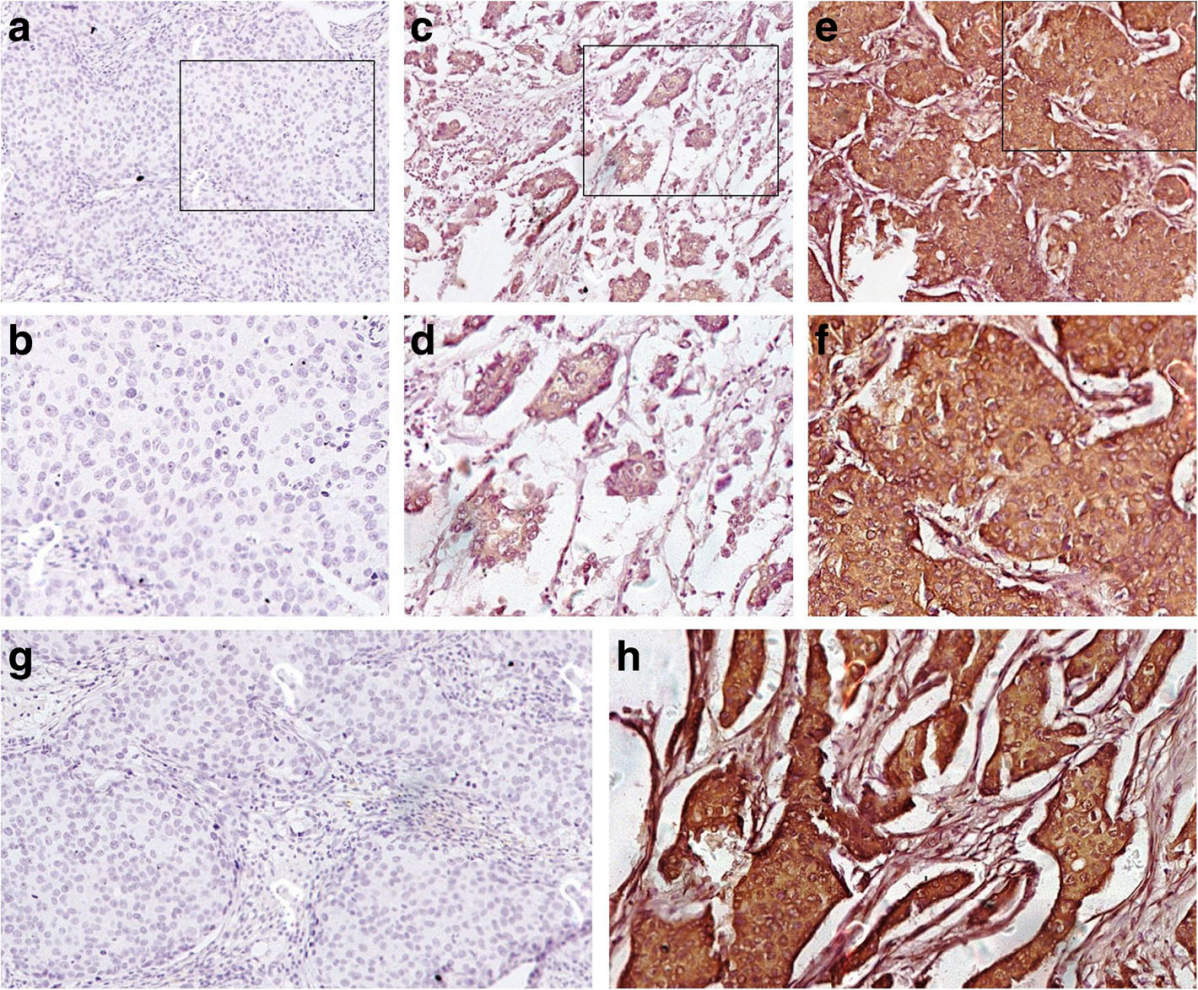
Representative immunohistochemical staining for SNCG expression in breast cancer tissues. **a** 100 × and **b** 200 × staining of negative sample 1 (area grade 0, intensity grade 0); **c** 100 × and **d** 200 × staining of negative sample 2 (area grade 1, intensity grade 1); **e** 100 × and **f** 200 × staining of positive sample (area grade 1, intensity grade 3); **g** Staining of negative control (100×); **h** Staining of positive control (100×)

IHC staining of SNCG was performed for all samples. According to our grading criteria, 139 samples among 238 were defined as SNCG negative while another 99 were defined as SNCG positive (total positive rate = 41.6 %). Representative images of SNCG staining in breast cancer tissues with examples of scoring were shown in Fig. 1. Positive rates of SNCG were 41.5 % (34/82) in patients that received radiotherapy and 41.7 % (65/156) in those that did not receive radiotherapy, and there was no significant difference (*χ*^*2*^ = 0.001, *p* = 0.976). For patients treated with radiotherapy or not, there were no significant associations between SNCG expression and Age (*p* = 0.767, 0.665), Tumor size (*p* = 0.145, 0.142), Metastasis lymph node (*p* = 0.117, 0.332), TNM stage (*p* = 0.428, 0.957), ER status (*p* = 0.304, 0.998), PR status (*p* = 0.171, 0.904), or HER2 status (*p* = 0.351, 0.646), and all of the clinicopathologic features in both subgroups were equally distributed (Table 1).

**Table 1:** Association of SNCG expression with clinicopathological parameters in breast cancer patients were or were not treated with radiotherapy

Characteristics	Radiotherapy				No Radiotherapy			
	SNCG-	SNCG+	*χ* ^*2*^	*p*-value	SNCG-	SNCG+	*χ* ^*2*^	*p*-value
Age			0.088	0.767			0.188	0.665
<50 years	21	16			43	33		
≥50 years	27	18			48	32		
Tumor size			2.126	0.145			2.161	0.142
<2 cm	29	15			40	21		
≥2 cm	19	19			51	44		
Metastasis lymph node			4.393^a^	0.117			2.202	0.332
0	19	7			34	17		
1–3	6	3			31	27		
≥4	23	24			26	21		
TNM stage			0.628	0.428			0.003^a^	0.957
I	12	6			7	4		
II, III	36	28			84	61		
ER			1.058	0.304			0.001	0.998
negative	12	13			34	25		
positive	32	21			53	39		
PR			1.874	0.171			0.014	0.904
negative	23	23			44	33		
positive	21	11			43	31		
HER2			0.871	0.351			0.211	0.646
negative	28	21			50	41		
positive	7	9			14	14		

^a^Chi-square test with Yates’ continuity correction

### Relationship between SNCG expression and radiotherapy stratified survival

**Fig. 2:**
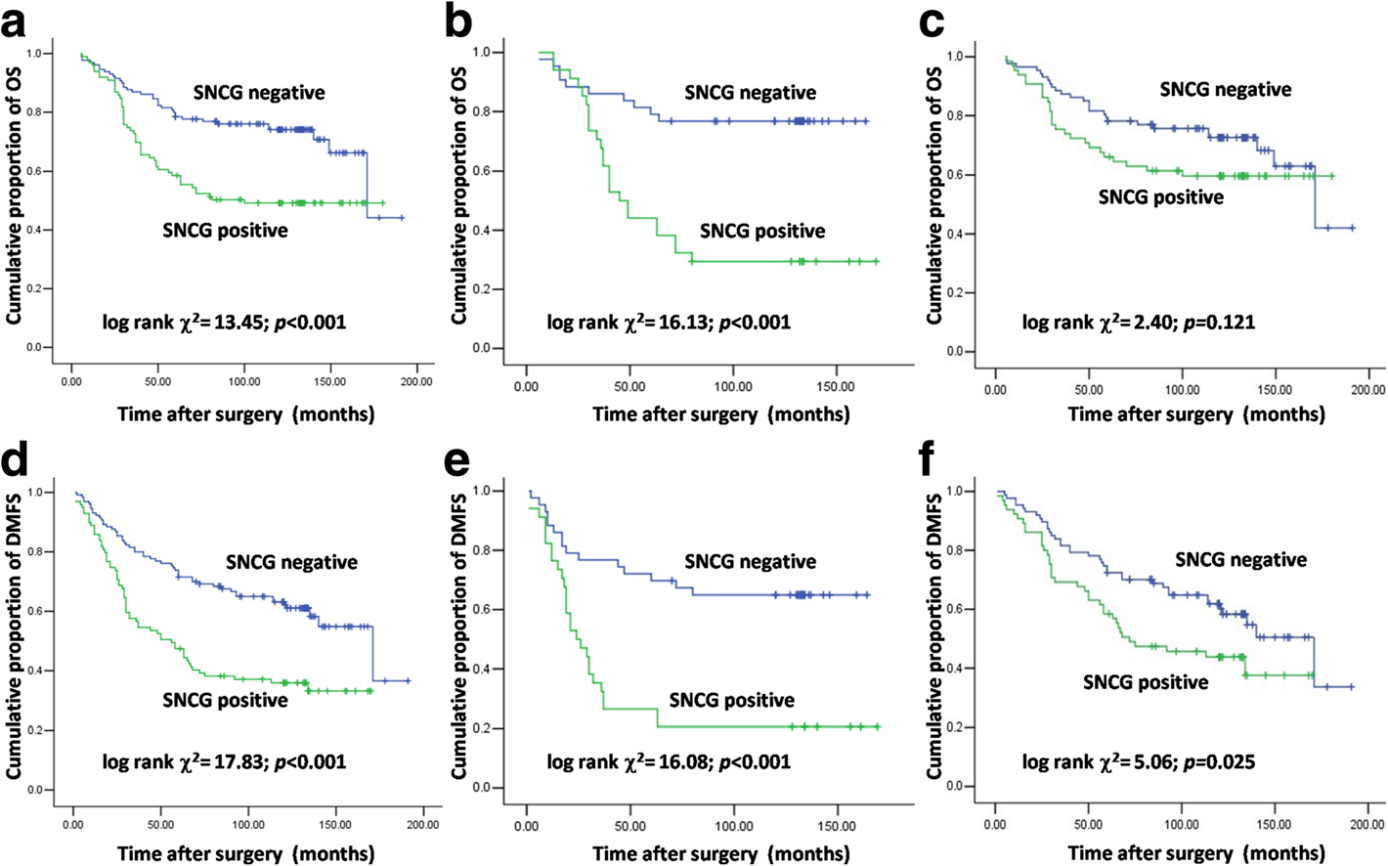
Kaplan-Meier curve of OS/DMFS in breast cancer patients evaluated according to SNCG expression, stratified with radiotherapy reception. All patients (**a**, **d**); patients received radiotherapy (**b**, **e**); patients did not receive radiotherapy (**c**, **f**)

Positive SNCG was correlated with decreased OS (median OS: 108.3 vs. 144.6 months; log rank *χ*^*2*^ = 13.45; *p* < 0.001; Fig. 2a) and DMFS (median DMFS: 81.2 vs. 127.7 months; log rank *χ*^*2*^ = 17.83; *p* < 0.001; Fig. 2d) in breast cancer patients, regardless of the utilization or non-utilization of radiotherapy.

Among patients that received radiotherapy, those with positive SNCG expression had a 55.0 months shorter median OS than those with negative SNCG expression (median OS: 78.4 vs. 133.4 months; log rank *χ*^*2*^ = 16.13; *p* < 0.001; Fig. 2b). However, among patients that were not subjected to radiotherapy, there was no significant difference between OS of patients with positive SNCG expression and those with negative SNCG expression (median OS: 122.4 vs. 143.1 months; log rank *χ*^*2*^ = 2.40; *p* = 0.121; Fig. 2c). Similar results were also obtained for DMFS (for patients received radiotherapy, median DMFS: 52.9 vs. 116.7 months, Fig. 2e; for patients did not receive radiotherapy, median DMFS: 95.1 vs. 126.7 months, Fig. 2f).

### Univariate and multivariate analysis for the radiotherapy stratified prognosis

In univariate analysis, tumor size, lymph nodes metastasis, TNM stage, SNCG expression were statistically associated with OS in patients that received radiotherapy, while lymph nodes metastasis, TNM stage, and HER2 status were prognostic factors of OS in patients that did not receive radiotherapy (Table 2).

**Table 2:** Prognostic factors of OS in univariate analysis of breast cancer patients were or were not treated with radiotherapy

Characteristics	Radiotherapy		No Radiotherapy	
	RR (95 % CI)	*p*-value	RR (95 % CI)	*p*-value
Age		0.429		0.739
≥50 vs. <50	0.759 (0.383, 1.503)		1.098 (0.635, 1.898)	
Tumor size		0.011		0.730
≥2 cm vs. <2 cm	2.432 (1.203, 4.918)		1.104 (0.628, 1.944)	
Metastasis lymph node		<0.001		0.001
1-3 vs. 0	1.878 (0.314, 11.243)		0.935 (0.433, 2.017)	
≥4 vs. 0	8.151 (2.475, 26.845)		2.763 (1.415, 5.396)	
TNM stage		<0.001		<0.001
II, III vs. I	6.624 (2.556, 17.171)		2.906 (1.681, 5.025)	
SNCG		<0.001		0.126
Positive vs. Negative	4.058 (1.929, 8.537)		1.530 (0.888, 2.638)	
ER		0.176		0.183
Positive vs. Negative	0.613 (0.306, 1.226)		1.530 (0.818, 2.861)	
PR		0.485		0.232
Positive vs. Negative	0.782 (0.391, 1.561)		1.423 (0.798, 2.538)	
HER2		0.985		0.018
Positive vs. Negative	1.008 (0.434, 2.341)		2.304 (1.152, 4.606)	

*RR* Risk Ratio, *CI* confidence interval

Multivariate analyses using COX regression analysis identified TNM stage (Wald *χ*^*2*^ = 10.31; *p* = 0.001) and SNCG (Wald *χ*^*2*^ = 6.62; *p* = 0.010) expression, which were both independent predictors of OS in patients that received radiotherapy. However, in patients that did not receive radiotherapy, only TNM stage (Wald *χ*^*2*^ = 7.32; *p* = 0.007) remained an independent prognostic factor (Table 3). Similar results were also obtained for DMFS (Additional file 1: Table S1 and S2). Taken together, SNCG expression affected the survival of breast cancer patients to a greater extent in patients that received radiotherapy.

**Table 3:** Independent predictors of OS in multivariate analysis of breast cancer patients were or were not treated with radiotherapy

Characteristics	Radiotherapy		No Radiotherapy	
	RR (95 % CI)	*p*-value	RR (95 % CI)	*p*-value
TNM stage		0.001		0.007
II, III vs. I	4.960 (1.866, 13.183)		2.548 (1.294, 5.019)	
SNCG		0.010		
Positive vs. Negative	2.726 (1.270, 5.850)			

### Association between SNCG expression and radiation stress response gene sets

SMIRNOV_RESPONSE_TO_IR_2HR_DN gene set includes a series of genes that are down-regulated in lymphocytes at 2 h after exposure to 10 Gy dose of ionizing radiation [31] and MAYBURD_RESPONSE_TO_L663536_DN gene set includes a series of genes down-regulated in cancer cells after treatment with L663536, a small molecular chemical found to enhance the effect of radiation in cancer cells [32]. These two gene sets located in the Molecular Signatures Database (MSigDB) were downloaded to perform GSEA analysis in online datasets *E-TABM-158* and *GSE1456*.

**Fig. 3:**
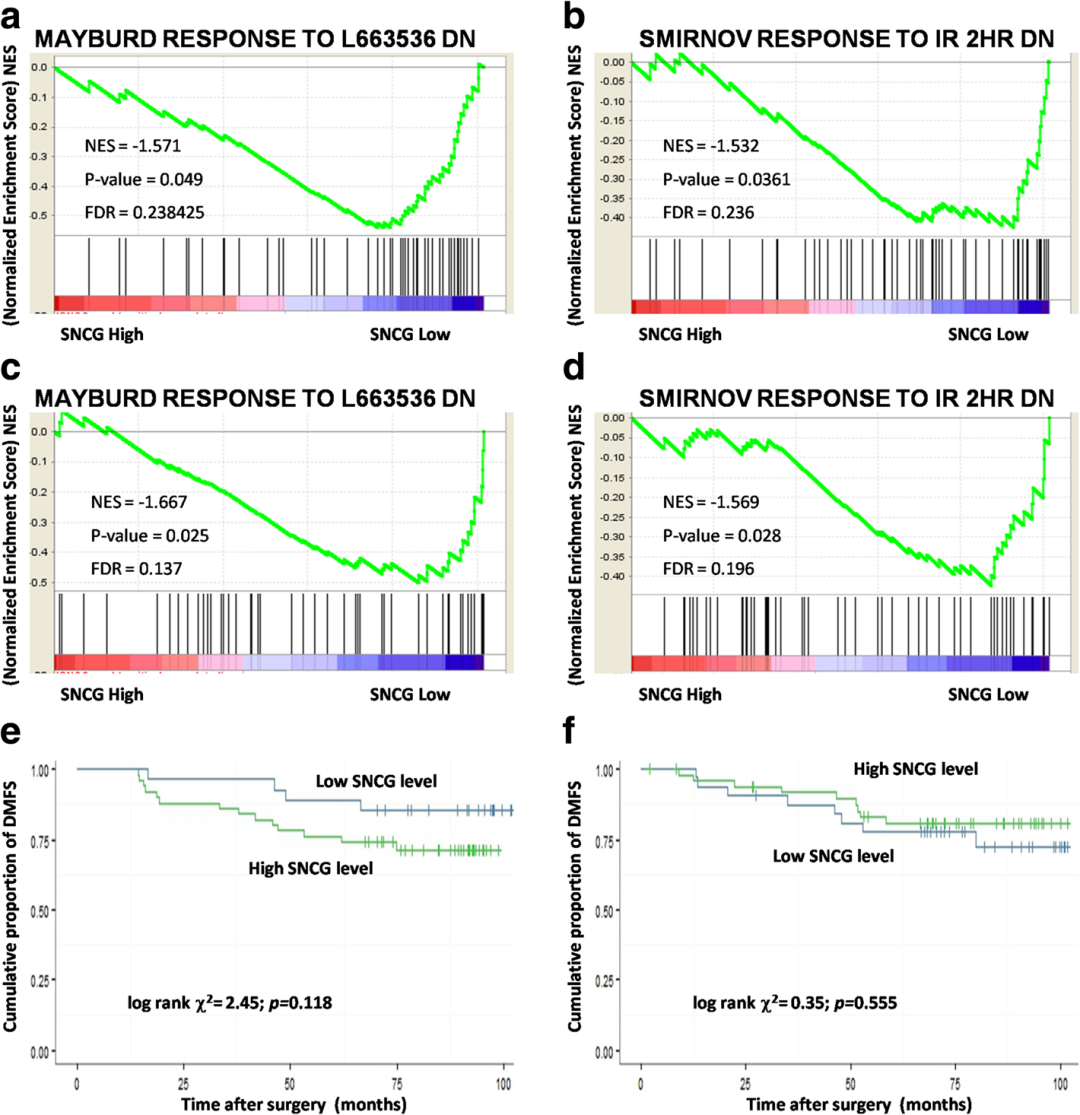
SNCG expression is associated with radiation related GSEA gene sets and radiotherapy related survival. Normalized Enrichment Score (NES) of MAYBURD_RESPONSE_TO_L663536_DN gene set in breast cancer dataset *E-TABM-158* (**a**) and *GSE1456* (**c**); Normalized Enrichment Score (NES) of SMIRNOV_RESPONSE_TO_IR_2HR_DN gene set in breast cancer dataset *E-TABM-158* (**b**) and *GSE1456* (**d**); Kaplan-Meier curve of DMFS evaluated according to SNCG expression in patients received radiotherapy (**e**) and patients did not receive radiotherapy (**f**) in breast cancer dataset *E-TABM-158*

In ArrayExpress dataset *E-TABM-158*, the Normalized Enrichment Score (NES) of MAYBURD_RESPONSE_TO_L663536_DN gene set was −1.571 (*p* = 0.049, FDR = 0.238, Fig. 3a), indicating SNCG expression is negatively correlated with genes in this gene set significantly. For SMIRNOV_RESPONSE_TO_IR_2HR_DN gene set, a NES of −1.532 was achieved (*p* = 0.036, FDR = 0.236, Fig. 3b).

Similar results were also obtained in the NCBI GEO dataset *GSE1456*. NES of MAYBURD_RESPONSE_TO_L663536_DN was −1.667 (*p* = 0.025, FDR = 0.137, Fig. 3c), while NES of MIRNOV_RESPONSE_TO_IR_2HR_DN was −1.569 (*p* = 0.028, FDR = 0.196, Fig. 3d).

### Relationship between SNCG expression and radiotherapy stratified survival in validating dataset

A validating survival analysis was then conducted. Considering that the sample size of *GSE1456* was too small to stratify, only the *E-TABM-158* dataset was used in the subsequent survival analyses. The *E-TABM-158* dataset was stratified to the radiotherapy subgroup and the non-radiotherapy subgroup.

In the radiotherapy subgroup, patients with high SNCG levels had a worse DMFS values than those with low SNCG levels. A suggestive *p*-value was achieved, considering the small sample size (Fig. 3e, log-rank *χ*^*2*^ = 2.45, *p* = 0.118). In the non-radiotherapy subgroup, patients with different SNCG levels had nearly the same survival outcome (Fig. 3f, log-rank *χ*^*2*^ = 0.35, *p* = 0.555). Additionally, the percentage of censored data was too high to calculate the median DMFS in both subgroups.

**Fig. 4:**
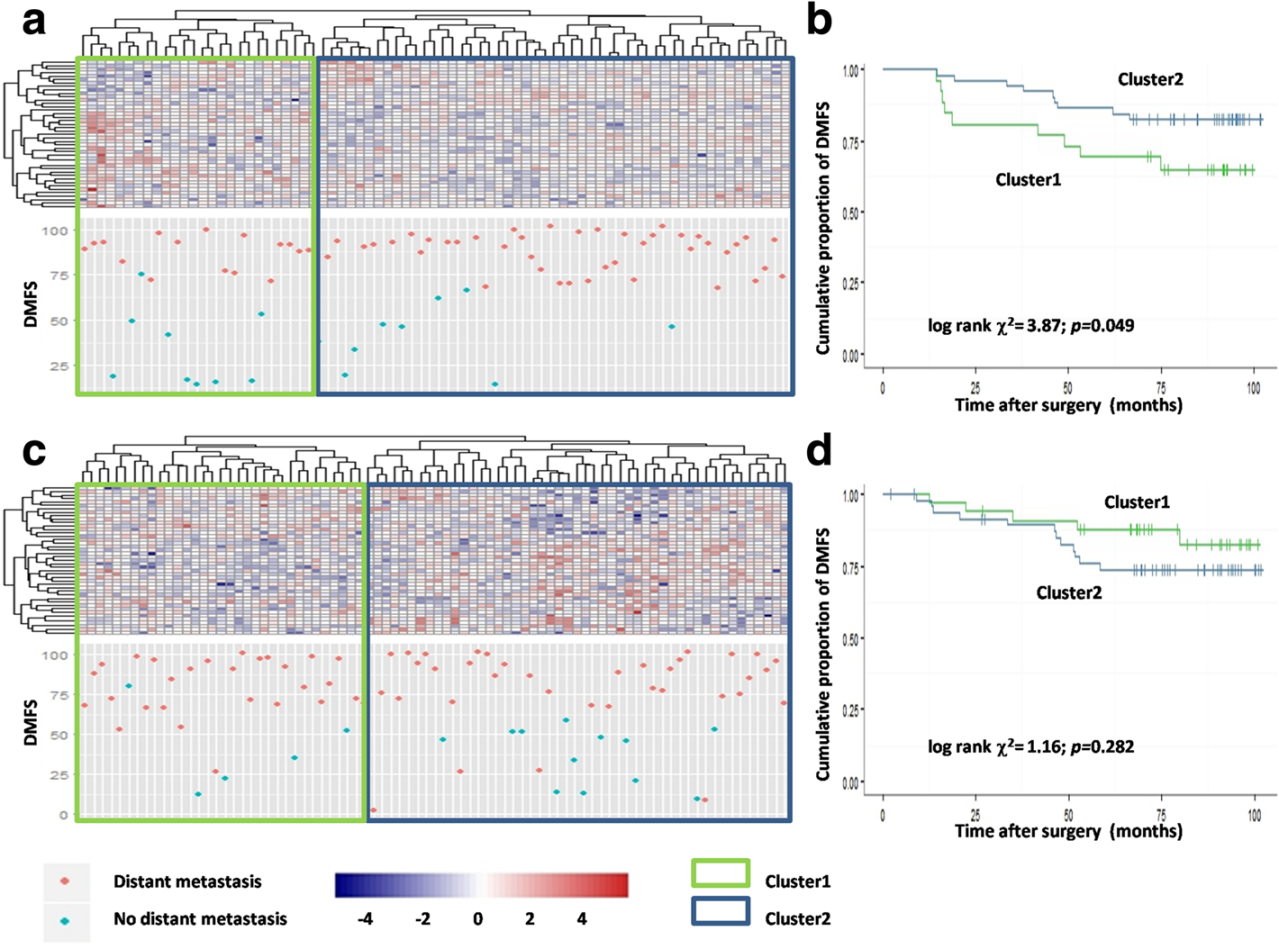
Relationship between expressions of SNCG correlated genes and radiotherapy stratified survival in validating dataset. Hierarchical clustering result of patients received radiotherapy (**a**) and patients did not receive radiotherapy (**c**) in breast cancer dataset *E-TABM-158* by SNCG-correlated genes; Kaplan-Meier curve of DMFS in patients received radiotherapy (**b**) and patients did not receive radiotherapy (**d**), grouped by clustering result of SNCG-correlated genes, in breast cancer dataset *E-TABM-158*

To get a survival indicator of higher resolution, a set of SNCG correlated genes (the expression of those genes was correlated with SNCG in both *E-TABM-158* and *GSE1456* datasets) were recruited to construct a novel SNCG signature. In the radiotherapy subgroup, patients with different SNCG signatures showed a significant difference in DMFS (log-rank *χ*^*2*^ = 3.87, *p* = 0.049, Fig. 4a, b), while in the non-radiotherapy subgroup, patients with different SNCG signatures had similar DMFS (log-rank *χ*^*2*^ = 1.16, *p* = 0.282, Fig. 4c, d).

### Relationship between SNCG expression and radiotherapy stratified survival in other types of cancer

**Fig. 5:**
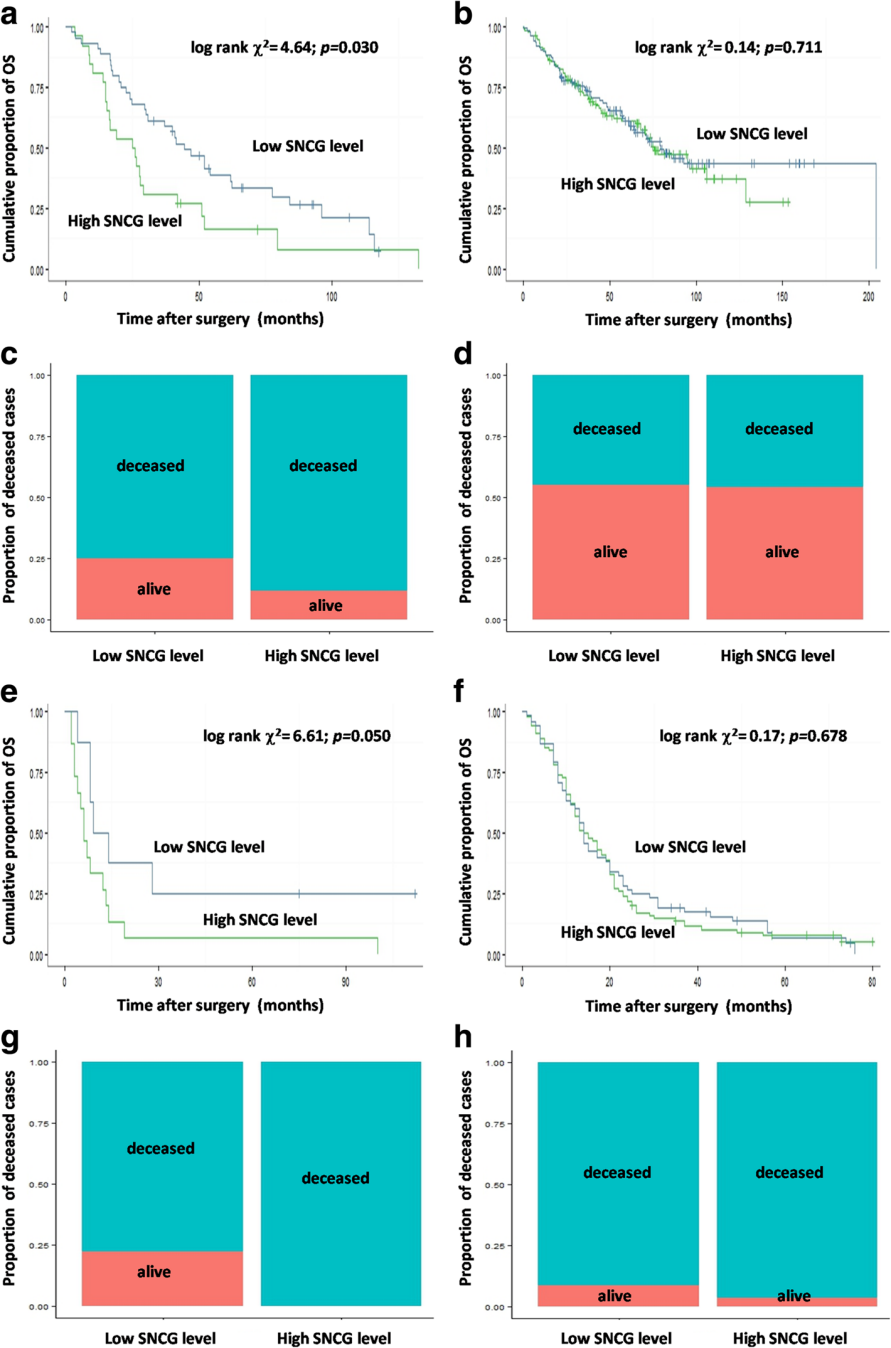
SNCG expression is associated with radiotherapy related survival in other types of cancer. Kaplan-Meier curve of OS in patients received radiotherapy (**a**) and patients did not receive radiotherapy (**b**) in lung cancer dataset *CaArray*; Mortal rate of patients received radiotherapy (**c**) and patients did not receive radiotherapy (**d**) in lung cancer dataset *CaArray*; Kaplan-Meier curve of OS in patients received radiotherapy (**e**) and patients did not receive radiotherapy (**f**) in glioblastoma dataset *GSE13041*; Mortal rate of patients received radiotherapy (**g**) and patients did not receive radiotherapy (**h**) in glioblastoma dataset *GSE13041*

Additionally, a relationship between SNCG expression and radiotherapy-related survival was further testified in other cancers. Pertaining to the lung cancer dataset *CaArray*, high SNCG levels indicated a significantly worse OS than low SNCG level in patients that received radiotherapy (median OS: 25.5 vs. 44.4 months; log rank *χ*^*2*^ = 4.64; *p* = 0.030; Fig. 5a, c). Similar OS was observed regardless of the SNCG level in patients that did not receive radiotherapy (median OS: 75.7 vs. 79.5 months; log rank *χ*^*2*^ = 0.14; *p* = 0.711; Fig. 5b, d).

Regarding the glioblastoma dataset *GSE13041*, a high SNCG level indicated a marginally significantly worse OS than low SNCG level in patients received radiotherapy (median OS: 6.0 vs. 11.5 months; log rank *χ*^*2*^ = 3.61; *p* = 0.050; Fig. 5e, g). In patients that did not receive radiotherapy, a similar OS was observed regardless of the SNCG level (median OS: 14.5 vs. 14.0 months; log rank *χ*^*2*^ = 0.17; *p* = 0.678; Fig. 5f, h).

## Discussion

Despite that the overexpression of SNCG has long been observed in cancers and high levels of SNCG expression has been validated to be associated with poorer OS and DMFS in multiple types of cancer, the suitability of SNCG expression as a biomarker for patient selection in radiotherapy remains largely unknown [17–21]. Kang et al. reported that radiotherapy induced SNCG expression in the MCF7 cell line, which may contribute to immune suppressive effects by inhibiting the differentiation and activation of dendritic cells. Yet the linkage between SNCG and radiotherapy remains to be elucidated [33]. In this study, to reveal the association between SNCG expression and the radiotherapeutic response, we performed a retrospective analysis in which 238 of 454 breast cancer patients were selected according to the indications for postoperative radiotherapy. The aim of this case-by-case screening was to reduce the heterogeneity of our study cohort. In both subgroups stratified with radiotherapy reception, no clinicopathologic feature was found to be associated with SNCG expression, indicating that our patients’ data from different subgroups were comparable. Meanwhile, none of our selected patients have received neoadjuvant chemotherapy, which we believe can minimize the pre-surgical bias between groups of patients. Overall, patients with positive SNCG expression status showed a significantly worse prognosis than patients with negative SNCG expression, which is consistent with the results of previous reports [17, 18].

Detailed analysis has revealed that among patients that have received radiotherapy, the diversity of prognosis between the SNCG positive and the SNCG negative groups was significantly expanded among patients without radiotherapy treatment. The negative impact of SNCG on prognosis were twice higher for radiotherapy treated patients than for those who did not receive radiotherapy (medium diversity of OS: −55.0 vs. -20.7 months; medium diversity of DMFS: −63.8 vs. -31.6 months), indicating SNCG is indeed associated with radiotherapeutic efficacy. A COX regression analyses was performed to rule out possible confounding factors. SNCG expression was identified as an independent predictor of OS in patients that received radiotherapy, but not in those that received no radiotherapy. The same analysis was also conducted only for patients of stage III and SNCG was again found to be associated with radiotherapy efficacy (data not shown). Taken together, we hypothesize that positive SNCG may indicate a lack of benefit from radiotherapy for breast cancer patients.

To test whether SNCG expression was correlated with cell response to radiation, two radiation stress related gene sets (SMIRNOV_RESPONSE_TO_IR_2HR_DN [31] and MAYBURD_RESPONSE_TO_L663536_DN [32]) were simultaneously used for gene set enrichment analysis (GSEA) in online breast cancer datasets *E-TABM-158* and *GSE1456*. In both datasets, SNCG expression is negatively enriched with both gene sets, indicating that SNCG was highly correlated with radiation stress. To further elucidate SNCG’s association with radiotherapy, the online dataset *E-TABM-158* was used for stratified survival analysis. In the radiotherapy subgroup, a high-SNCG expression was suggested to be related with adverse DMFS despite that the statistic was not significant. SNCG and SNCG-correlated genes were recruited to build a signature, and signature related prognosis was observed. In contrast, in the non-radiotherapy subgroup, prognosis showed no difference whether grouped by a SNCG or a SNCG-correlated signature. The results presented here strongly support that positive SNCG may serve as a potential biomarker to identify breast cancer patients who are less likely to benefit from radiotherapy.

How SNCG expression affects sensitivity to radiotherapy still remains unclear. In breast cancer cells, SNCG interacts with phospholipase Cβ2 to modulate G protein activation [34] and it also has the potential to stimulate the ER pathway [35], which may increase cell malignancy. Additionally, SNCG confers resistance to anti-microtubule agents by forming a complex with BubR1 [11, 12, 36]. Considering that the main mechanism of radiotherapy is DNA damage-induced cell cycle arrest and growth inhibition [37], interaction between SNCG and BubR1 may partially explain the role of SNCG in breast cancer cells’ response to radiation. Given that radiation could also kill cancer cells effectively through facilitating Reactive Oxygen Species (ROS) generation [38], the possible association between SNCG and ROS-regulated signaling pathway is worth further investigation.

We also explored the possibility that SNCG may serve as a radiotherapy-related biomarker in other types of cancer. The lung cancer dataset *CaArray* and the glioblastoma dataset *GSE13041* were recruited and results similar to breast cancer were achieved, suggesting that SNCG’s role in identifying cancer patients less likely to benefit from radiotherapy may be universal in various types of cancer. To make this suggestion stronger, datasets with enough radiotherapy information of more cancer types were needed.

## Conclusion

This is the first study that demonstrates that SNCG could potentially be used as a biomarker to predict a worse patient outcome and less patient benefit as a consequence of radiotherapy for breast cancer. This study also raises an important issue regarding the postoperative adjuvant management of breast cancer. We truly believe that this is an important analysis since patients who have recently undergone a mastectomy and have begun receiving radiotherapy will derive the most benefit from the appropriate risk evaluation and treatment plan selection. Furthermore, an early prediction of radiotherapy treatment efficacy could also potentially improve their quality of life. Considering our results were obtained from a retrospective study, confirmative studies with other cohorts or by prospective, randomly controlled, and double-blinded studies were required.

## Additional file

Additional file 1:
**Table S1.** Prognostic factors of DMFS in univariate analysis of breast cancer patients were or were not treated with radiotherapy. **Table S2.** Independent predictors of DMFS in multivariate analysis of breast cancer patients were or were not treated with radiotherapy. (DOC 60 kb)

## Abbreviations

CI: Confidence interval
DAB: Diaminobenzidine
DMFS: Distant metastasis-free survival
ER: Estrogen receptor
FDR: False discovery rate
GSEA: Gene set enrichment analysis
HR: Hazard ratios
IHC: Immunohistochemistry
MSigDB: Molecular signatures database
NES: Normalized enrichment score
OS: Overall survival
ROS: Reactive oxygen species
SNCG: Synuclein-γ
