# Supplementary material for: Overexpression of synuclein-γ predicts lack of benefit from radiotherapy for breast cancer patients
Source: BMC Cancer. 2016 Sep 5;16(1):717. doi: 10.1186/s12885-016-2750-y (PMC5011985; doi:10.1186/s12885-016-2750-y)
Supplement: Additional file 1: — Table S1. Prognostic factors of DMFS in univariate analysis of breast cancer patients were or were not treated with radiotherapy. Table S2. Independent predictors of DMFS in multivariate analysis of breast cancer patients were or were not treated with radiotherapy. (DOC 60 kb) [file 12885_2016_2750_MOESM1_ESM.doc]

**Supplementary Table 1. Prognostic factors of DMFS in univariate analysis of breast cancer patients were or were not treated with radiotherapy.**

| Characteristics | Radiotherapy | |  | No Radiotherapy | |
| --- | --- | --- | --- | --- | --- |
|  | RR (95% CI) | *p*-value |  | RR (95% CI) | *p*-value |
| Age |  | 0.404 |  |  | 0.360 |
| ≥50 vs. ＜50 | 1.300 (0.702, 2.408) |  |  | 1.238 (0.783, 1.958) |  |
| Tumor size |  | 0.002 |  |  | 0.533 |
| ≥2 cm vs. ＜2 cm | 2.743 (1.456, 5.169) |  |  | 1.159 (0.729, 1.841) |  |
| Metastasis lymph node |  | <0.001 |  |  | 0.006 |
| 1-3 vs. 0 | 3.104 (0.626, 15.388) |  |  | 0.758 (0.416, 1.382) |  |
| ≥4 vs. 0 | 12.883 (3.936, 42.165) |  |  | 1.835 (1.067, 3.156) |  |
| TNM stage |  | <0.001 |  |  | 0.001 |
| Ⅲ vs.Ⅰ, Ⅱ | 11.126 (4.323, 28.637) |  |  | 2.140 (1.352, 3.387) |  |
| SNCG |  | <0.001 |  |  | 0.027 |
| Positive vs. Negative | 3.420 (1.799, 6.498) |  |  | 1.682 (1.061, 2.666) |  |
| ER |  | 0.014 |  |  | 0.992 |
| Positive vs. Negative | 0.461 (0.250, 0.852) |  |  | 1.002 (0.618, 1.626) |  |
| PR |  | 0.113 |  |  | 0.941 |
| Positive vs. Negative | 0.600 (0.319, 1.130) |  |  | 0.982 (0.612, 1.575) |  |
| HER2 |  | 0.986 |  |  | 0.353 |
| Positive vs. Negative | 0.993 (0.470, 2.099) |  |  | 1.339 (0.723, 2.479) |  |

**Supplementary Table 2. Independent predictors of DMFS in multivariate analysis of breast cancer patients were or were not treated with radiotherapy.**

| Characteristics | Radiotherapy | |  | No Radiotherapy | |
| --- | --- | --- | --- | --- | --- |
|  | RR (95% CI) | *p*-value |  | RR (95% CI) | *p*-value |
| TNM stage |  | <0.001 |  |  | 0.001 |
| Ⅲ vs.Ⅰ, Ⅱ | 9.267 (3.531, 24.323) |  |  | 2.119 (1.339, 3.355) |  |
| SNCG |  | 0.025 |  |  |  |
| Positive vs. Negative | 2.140 (1.102, 4.157) |  |  |  |  |
